# Ciclopirox activates ATR-Chk1 signaling pathway leading to Cdc25A protein degradation

**DOI:** 10.18632/genesandcancer.166

**Published:** 2018-01

**Authors:** Tao Shen, Hongyu Zhou, Chaowei Shang, Yan Luo, Yang Wu, Shile Huang

**Affiliations:** ^1^ Department of Biochemistry and Molecular Biology, Louisiana State University Health Sciences Center, Shreveport, LA, USA; ^2^ Feist-Weiller Cancer Center, Louisiana State University Health Sciences Center, Shreveport, LA, USA; ^3^ State Key Laboratory of Biotherapy / Collaborative Innovation Center of Biotherapy, West China Hospital, Sichuan University, Chengdu, Sichuan, P.R. China

**Keywords:** Ciclopirox, Cdc25A, ATR, Chk1, DNA damage

## Abstract

Ciclopirox olamine (CPX), an off-patent anti-fungal drug, has been found to inhibit the G_1_-cyclin dependent kinases partly by increasing the phosphorylation and degradation of Cdc25A. However, little is known about the molecular target(s) of CPX responsible for Cdc25A degradation. Here, we show that CPX induced the degradation of Cdc25A neither by increasing CK1α or decreasing DUB3 expression, nor via activating GSK3β, but through activating Chk1 in rhabdomyosarcoma (Rh30) and breast carcinoma (MDA-MB-231) cells. This is strongly supported by the findings that inhibition of Chk1 with TCS2312 or knockdown of Chk1 profoundly attenuated CPX-induced Cdc25A degradation in the cells. Furthermore, we observed that CPX caused DNA damage, which was independent of reactive oxygen species (ROS) induction, but related to iron chelation. CPX treatment resulted in the activation of ataxia telangiectasia mutated (ATM) and ATM-and RAD3-related (ATR) kinases. Treatment with Ku55933 (a selective ATM inhibitor) failed to prevent CPX-induced Chk1 phosphorylation and Cdc25A degradation. In contrast, knockdown of ATR conferred high resistance to CPX-induced Chk1 phosphorylation and Cdc25A degradation. Therefore, the results suggest that CPX-induced degradation of Cdc25A is attributed to the activation of ATR-Chk1 signaling pathway, a consequence of iron chelation-induced DNA damage.

## INTRODUCTION

Ciclopirox olamine (CPX), which has a broad spectrum of action against dermatophytes, yeast, filamentous fungi and bacteria [1], has been widely used for the treatment of superficial fungal infection for over 20 years [2]. Recent studies have implicated that CPX also has potent anticancer activity, by inhibiting cell proliferation and inducing cell death in tumor cells [3-15]. In addition, CPX has been found to inhibit angiogenesis [16], although this remains disputable [17]. Moreover, CPX inhibits lymphangiogenesis [18]. Acute toxicity tests have shown that the oral LD_50_ values of CPX in rats, mice and rabbits are in the range of 1700-3290 mg/kg of body weight [19, 20], suggesting that CPX is well tolerated in the animals. Oral administration of CPX at 30 mg/kg body weight for 4 weeks or at 10 mg/kg for 3 months has not been found to exhibit obvious toxic symptoms (e.g. gross organ toxicity and body weight loss), in a variety of experimental animals [19], indicating a favorable systematic therapeutic index of CPX. Pharmacokinetics studies have demonstrated that ~10 µM serum concentrations of CPX are achievable after repeated administration of CPX to rats and dogs [19], with a half-life (t_1/2_) of 6.8-7.6 h [21]. Recently, a phase I clinical trial study has shown that oral administration of CPX at a dose of 40 mg/m^2^ once daily for 5 days is well tolerated in patients, and induces disease stabilization and/or hematologic improvement in 2/3 patients with advanced hematologic malignancies [22]. Hence, CPX has emerged as a new and promising anticancer agent.

Cyclin-dependent kinases (CDKs) play a key role in the regulation of cell cycle progression, and eventually cell division or cell proliferation [23]. The activities of CDKs are precisely regulated by multiple events such as phosphorylation, dephosphorylation and protein-protein interaction [23], among which, the removal of inhibitory phosphorylation on CDKs by cell division cycle 25 (Cdc25), a dual-specificity protein phosphatase, is critical to full activation of CDKs [23, 24]. Cdc25 family has three members: Cdc25A, Cdc25B, and Cdc25C [25]. Although the catalytic domains of these phosphatases are well conserved, their regulatory domains, which decide their subcellular distribution and turnover, are greatly diverse [25, 26]. Both Cdc25B and Cdc25C promote G_2_/M progression by primarily dephosphorylating CDK1 at T14/Y15, two inhibitory phosphorylation sites [27], whereas Cdc25A plays a pivotal role in assisting both G_1_/S and G_2_/M progression by dephosphorylating CDK4 at Y17 [28], CDK6 at Y24 [29], as well as CDK2 and CDK1 at T14/Y15 [30, 31]. As overexpression of Cdc25A predicts the malignancy and poor prognosis in cancer patients [25], Cdc25A has emerged as a new target for cancer therapy. Recent studies have shown that CPX, at high concentrations (> 10 µM), downregulates the cellular protein expression of cyclins (A, B1, D1 and E) and cyclin-dependent kinases (CDK2 and CDK4), and upregulates the expression of the CDK inhibitor p21^Cip1^ [5]. However, CPX, at low concentrations (≤5 µM), drastically reduces the cellular protein level of Cdc25A, which results in increased inhibitory phosphorylation of G_1_-CDKs, leading to the accumulation of cells in G_1_ phase of the cell cycle in tumor cells [32]. Furthermore, CPX neither alters the mRNA level of Cdc25A, nor reduces the protein synthesis of Cdc25A, but promotes the degradation of Cdc25A [32]. To better understand the molecular mechanism of anticancer action of CPX, it is of great importance to elucidate how CPX induces Cdc25A protein degradation.

The protein degradation of Cdc25A is primarily associated with DNA damage [33]. In response to genotoxic stress-induced DNA damage, ataxia telangiectasia mutated (ATM)/ATM-and RAD3-related (ATR)-Chk1/Chk2 cascade is activated, leading to Cdc25A/C degradation [34, 35], which stops cell cycle progression, and lets cells repair damaged DNA for survival [36]. Activated Chk1 phosphorylates S76 on Cdc25A, priming the further phosphorylation on S79 and S82, by casein kinase 1α (CK1α) [37-40]. The phosphorylation of S82 facilitates Cdc25A ubiquitination and subsequent proteasome-mediated degradation [40, 41], inhibiting CDK2 activity [42] and arresting cells at G_1_ phase of the cell cycle [43]. Besides, S76 residue of Cdc25A can also be phosphorylated by glycogen synthase kinase 3β (GSK3β) [44]. Chk2, another checkpoint protein activated by ATM [45], can phosphorylate Cdc25A at S124, which promotes Cdc25A degradation and downregulates CDK2 activity resulting in blockage of DNA synthesis [45]. This study sought to determine whether CPX-induced phosphorylation and degradation of Cdc25A is through activating CK1α, GSK3β, and/or ATM/ATR-Chk1/2 in tumor cells.

## RESULTS

### CPX-induced Cdc25A degradation is not attributed to increased CK1α or decreased DUB3 expression

**Figure 1:**
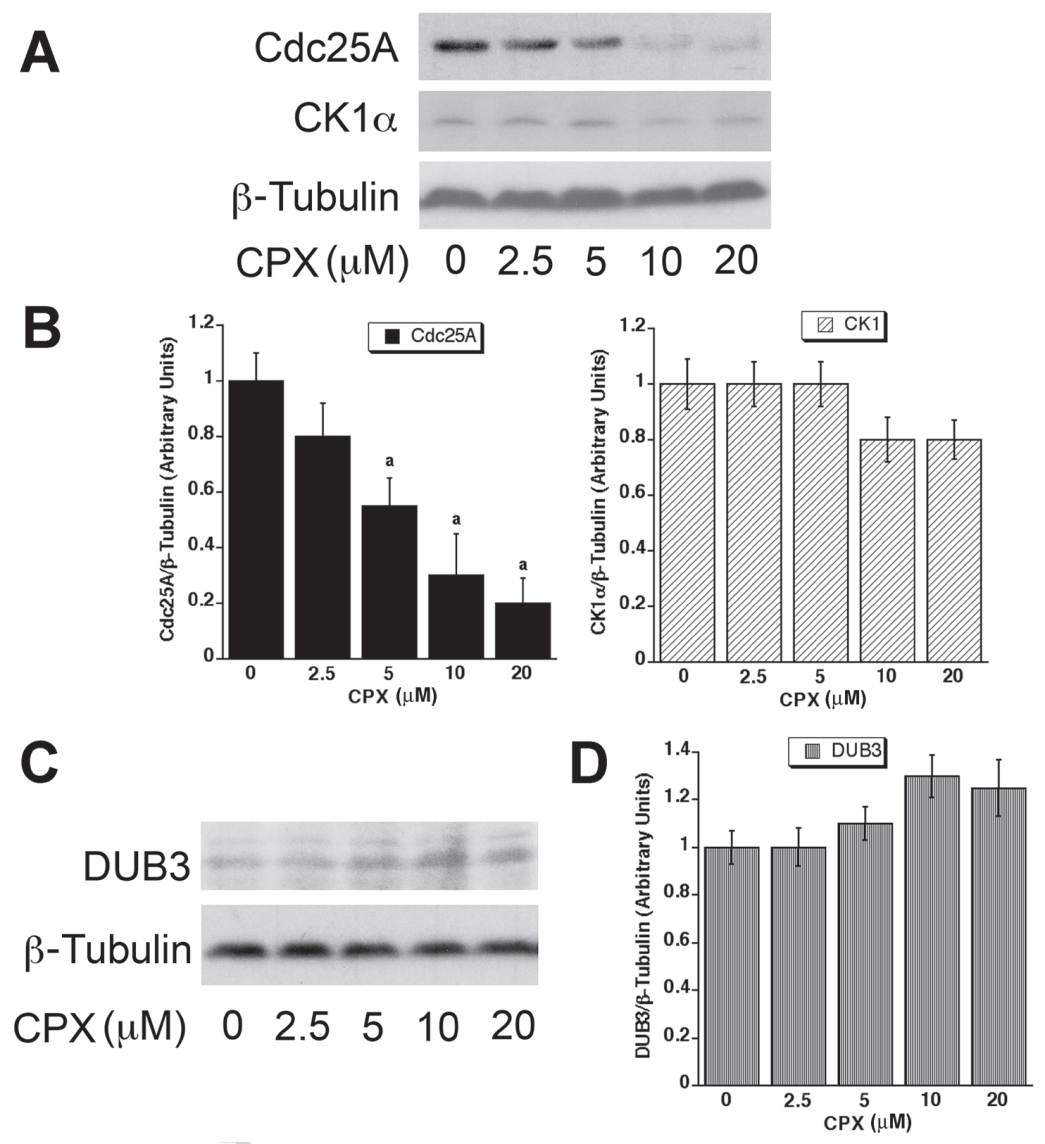
CPX neither upregulates CK1α, nor downregulates DUB3 expression (**A-D)** MDA-MB-231 cells were treated with CPX (0-20 μM) for 24 h, followed by Western blotting with indicated antibodies. β-tubulin was used for loading control. Densitometry for the bands in (**A)** and (**C)** was performed using NIH ImageJ, as shown in (**B)** and (**D)** respectively. Results are the means SE and are pooled from three independent experiments. ^*a*^, *P* < 0.05, difference *verse* control (0 μM CPX).

Our previous study has shown that CPX induces the phosphorylation of Cdc25A on S82, and Cdc25A-S82A mutant is resistant to CPX-induced degradation [32], indicating that the phosphorylation on S82 is essential for CPX-induced Cdc25A degradation. Since CK1α has been reported to be responsible for phosphorylating Cdc25A on S82 [40], and CK1α is a constitutively active kinase, whose activity is primarily determined by the cellular protein level [46], we reasoned that CPX-induced phosphorylation of Cdc25A (S82) is through upregulating protein expression of CK1α. For this, MDA-MB-231 cells were treated with CPX (0-20 μM) for 24 h, followed by Western blot analysis. In line with our previous observation [32], 24-h treatment with CPX downregulated the protein level of Cdc25A in a concentration-dependent manner (Figures 1A and 1B). Surprisingly, CPX did not obviously increase cellular protein expression of CK1α; in fact, at high concentrations (10-20 μM) slightly but not significantly downregulated the protein level of CK1α (Figures 1A and 1B). Therefore, our results ruled out the possibility that CPX promotes Cdc25A degradation by increasing CK1α expression.

As DUB3, a Cdc25A-specific ubiquitin hydrolase, has been reported to protect Cdc25A from degradation by removing conjugated ubiquitin from Cdc25A [47], next, we examined whether CPX promotes Cdc25A degradation by decreasing expression of DUB3. For this, MDA-MB-231 cells were treated with CPX (0-20 μM) for 24 h, followed by Western blot analysis. As shown in Figures 1C and 1D, CPX did not reduce the protein level of DUB3. On the contrary, CPX slightly increased the expression of DUB3 in a concentration-dependent manner. Thus, the results suggest that CPX-induced Cdc25A degradation is not due to decreased expression of DUB3.

### CPX-induced Cdc25A degradation is not due to activation of GSK3β

**Figure 2:**
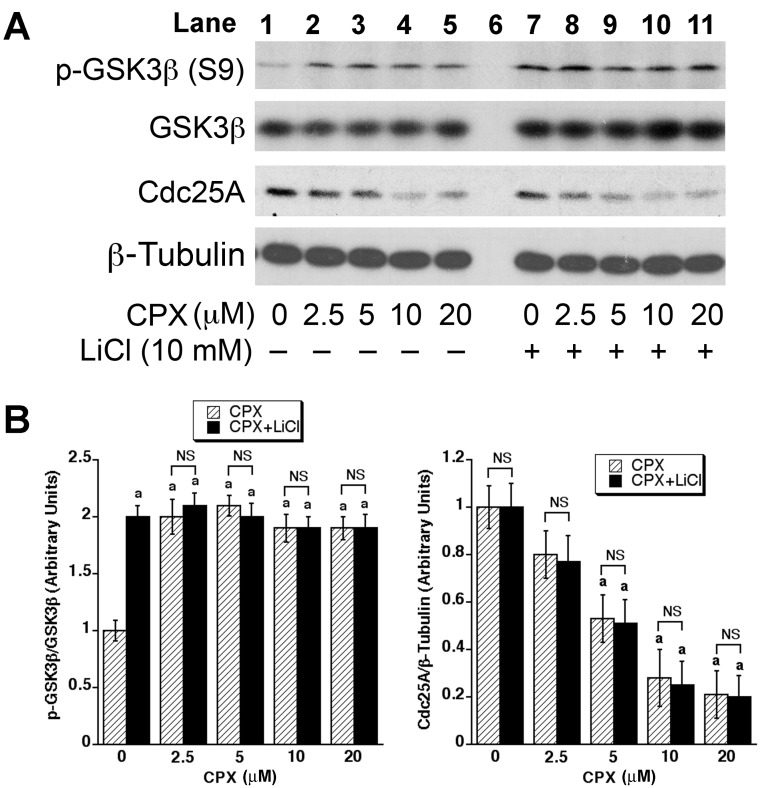
GSK3β is not involved in CPX-induced Cdc25A degradation (**A)** MDA-MB-231 cells were pretreated with or without LiCl (10 μM) for 2 h, and then incubated with CPX (0-20 μM) for 24 h, followed by Western blotting with indicated antibodies. β-tubulin was used for loading control. (**B)** Densitometry for the bands in (**A)** was performed using NIH ImageJ. Results are the means ± SE and are pooled from three independent experiments. ^*a*^, *P* < 0.05, difference *verse* control (0 μM CPX). NS, no significance.

The accumulation of Cdc25A phosphorylation on S82 can also result from the elevation of the phosphorylation on correspondent prime residues (e.g. S76) [37]. As GSK3β can phosphorylate Cdc25A on S76 [44], we wondered whether GSK3β is involved in CPX-induced Cdc25A degradation. For this, MDA-MB-231 cells were pretreated for 2 h with or without 10 mM of LiCl, an inhibitor of GSK3β [48], and then incubated with CPX (0-20 μM) for 24 h. As expected, treatment with LiCl elevated the phosphorylation level of GSK3β (S9) (Lane 7 *verse* Lane 1, Figure 2A), an inhibitory phosphorylation for GSK3β [49]. However, LiCl pretreatment did not prevent CPX-induced Cdc25A degradation (Figures 2A and 2B). Surprisingly, treatment with CPX alone increased the phosphorylation of GSK3β (S9) (Lanes 2-5 *verse* Lane 1, Figure 2A), indicating that CPX did not activate but actually inhibited the activity of GSK3β. Therefore, it is unlikely that CPX-induced Cdc25A degradation is mediated by GSK3β.

### CPX-induced Cdc25A degradation is related to activation of Chk1

**Figure 3:**
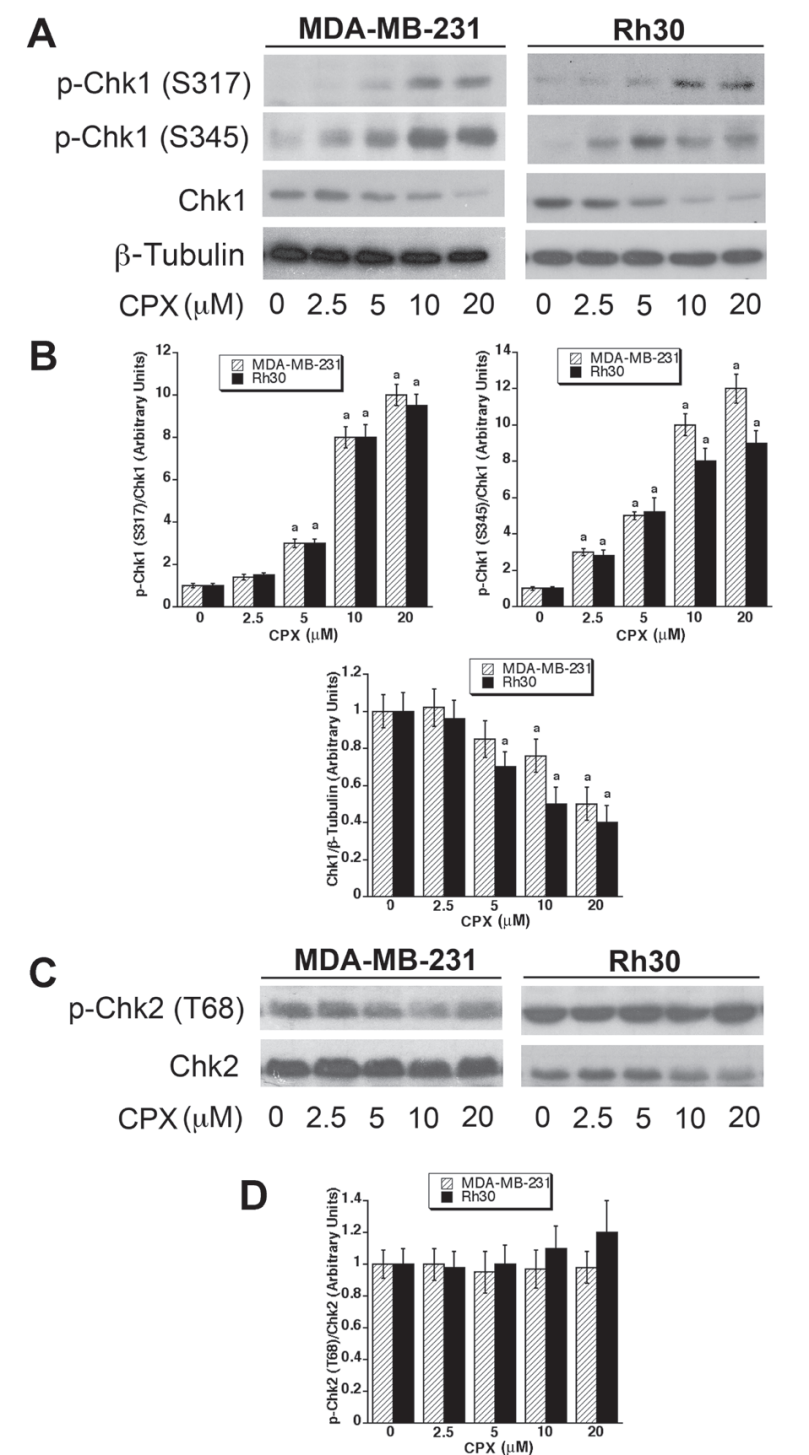
CPX induces activation of Chk1, but not Chk2 (**A-D)** MDA-MB-231 and Rh30 cells were treated with CPX (0-20 μM) for 24 h and 36 h, respectively, followed by Western blotting with indicated antibodies. β-tubulin was used for loading control. Densitometry for the bands in (**A)** and (**C)** was performed using NIH ImageJ, as shown in (**B)** and (**D)**, respectively. Results are the means ± SE and are pooled from three independent experiments. ^*a*^, *P* < 0.05, difference *verse* control (0 μM CPX).

As both Chk1 and Chk2 can phosphorylate Cdc25A on multiple residues, promoting SCF-mediated ubiquitination and proteolysis of Cdc25A [39, 45, 50-52], next, we asked whether CPX-induced Cdc25A degradation is mediated by Chk1 and/or Chk2. To this end, MDA-MB-231 cells were treated with CPX (0-20 μM) for 24 h, followed by Western blot analysis. As shown in Figures 3A and 3B, CPX (0-20 μM) treatment increased the phosphorylation on Chk1 (S317 and S345) in a concentration-dependent manner in MDA-MB-231 and Rh30 cells. Particularly, a robust phosphorylation on Chk1 was induced by 10 μM of CPX. Of note, treatment with 20 μM of CPX did not result in higher phosphorylation of Chk1, which might be associated with the drastically reduced level of the total Chk1 protein. On the contrary, treatment with CPX did not obviously alter the phosphorylation of Chk2 (T68) in the cells (Figures 3C and 3D). Therefore, the results indicate that Chk1, but not Chk2, is activated in the cells exposed to CPX.

**Figure 4:**
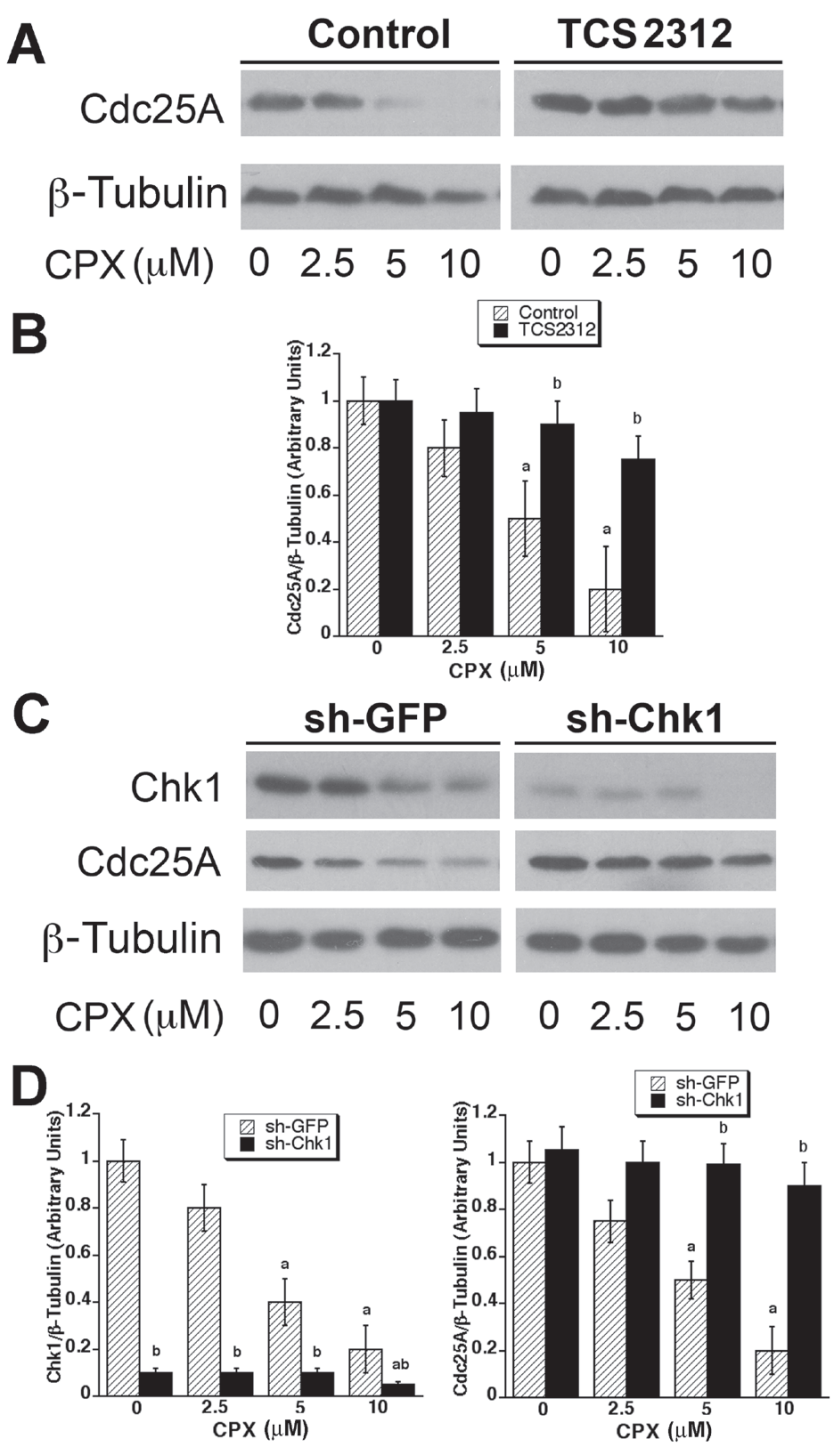
CPX-induced activation of Chk1 links to downregulation of Cdc25A (**A)** MDA-MB-231 cells were pretreated with or without TCS2312 (250 nM) for 2 h, and then incubated with CPX (0-10 μM) for 24 h, followed by Western blotting with indicated antibodies. β-tubulin was used for loading control. (**B)** Densitometry for the bands in (**A)** was performed using NIH ImageJ. Results are the means ± SE and are pooled from three independent experiments. ^*a*^, *P* < 0.05, difference *verse* control (0 μM CPX); ^*b*^, *P* < 0.05, difference between control and TCS2312 treatment. (**C)** MDA-MB-231 cells, infected with lentiviral shRNA to Chk1 or GFP (control), were incubated with CPX (0-10 μM) for 24 h, followed by Western blotting with indicated antibodies. β-tubulin was used for loading control. (**D)** Densitometry for the bands in (**C)** was performed using NIH ImageJ. Results are the means ± SE and are pooled from three independent experiments. ^*a*^, *P* < 0.05, difference *verse* control (0 μM CPX); ^*b*^, *P* < 0.05, difference between sh-GFP treatment and sh-Chk1 treatment.

To determine whether activation of Chk1 contributes to CPX-induced Cdc25A degradation, MDA-MB-231 cells were pretreated for 2 h with or without TCS2312 (250 nM), a selective inhibitor of Chk1 [53], and then incubated with CPX (0-10 μM) for 24 h, followed by Western blotting. Consistent with our previous finding [32], CPX (5-10 μM) alone obviously downregulated the expression of Cdc25A (Figures 4A and 4B). Interestingly, CPX-induced Cdc25A degradation was remarkably attenuated by TCS2312, suggesting that CPX-induced Cdc25A degradation may be attributed to activation of Chk1.

To confirm the above finding, RNA interference was employed. We found that lentiviral shRNA to Chk1 downregulated the protein expression of Chk1 in MDA-MB-231 cells by ~90%, compared with the control shRNA to GFP (Figures 4C and 4D), indicating that the shRNA to Chk1 was working well. As expected, MDA-MB-231 cells infected with the control shRNA to GFP (green fluorescence protein) were sensitive to CPX-induced Cdc25A degradation. In contrast, the cells infected with lentiviral shRNA to Chk1 were highly resistant to CPX-induced Cdc25A degradation (Figures 4C and 4D). Taken together, our results demonstrate that CPX induced-Cdc25A degradation is indeed due to activation of Chk1.

### ATR, but not ATM, is involved in mediating CPX-induced Cdc25A degradation

**Figure 5:**
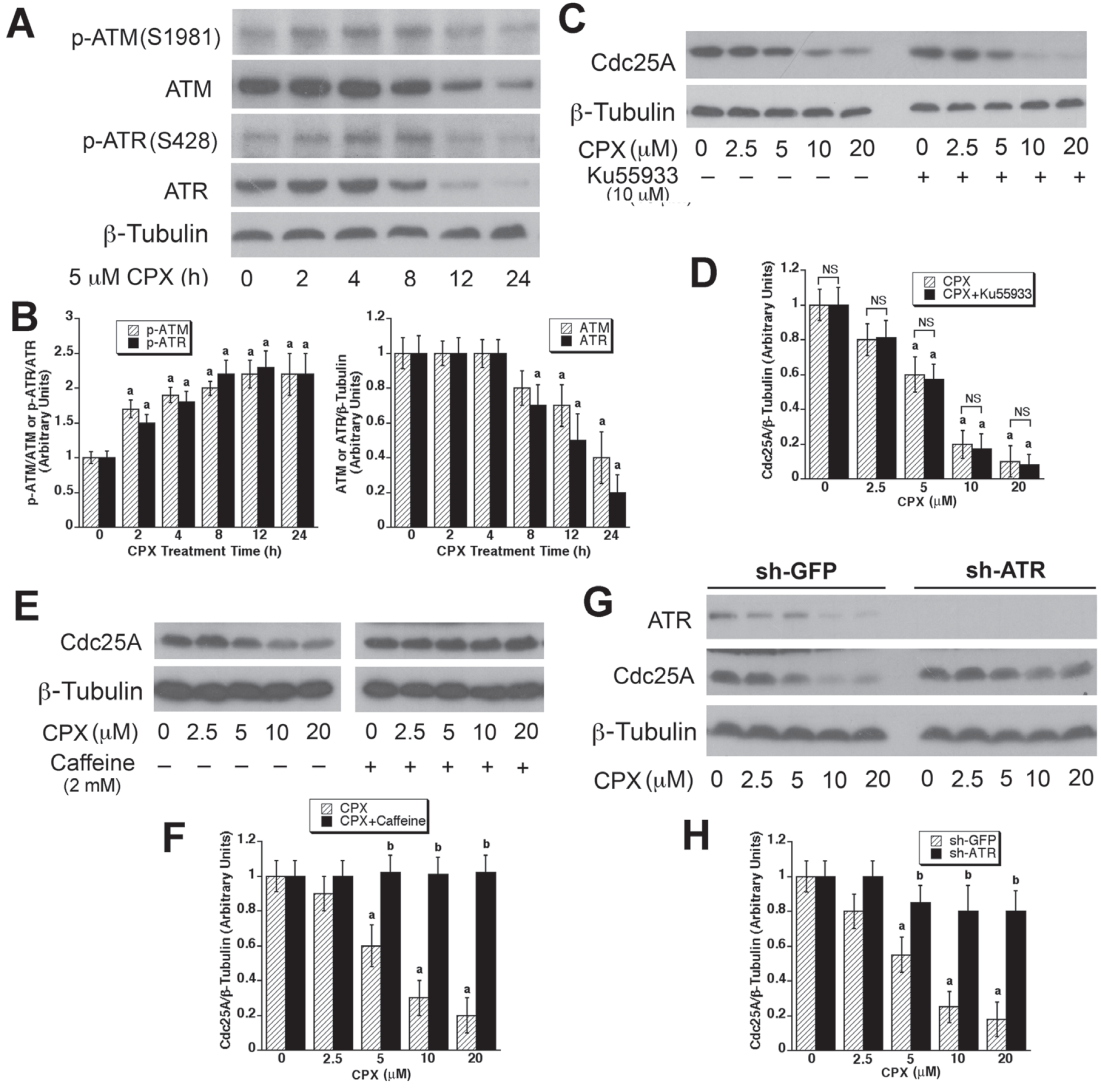
ATR, but not ATM, is involved in CPX-induced Cdc25A degradation **(A)** MDA-MB-231 cells were treated with CPX (5 μM) for 0-24 h, followed by Western blotting with indicated antibodies. β-tubulin was used for loading control. (**B)** Densitometry for the bands in (**A)** was performed using NIH ImageJ. Results are the means ± SE and are pooled from three independent experiments. ^*a*^, *P* < 0.05, difference *verse* control (CPX, 0 h). (**C**-**F)** MDA-MB-231 cells were pretreated with or without Ku55933 (10 μM) (**C)** or caffeine (2 mM) (**E)** for 2 h, and then incubated with CPX (0-20 μM) for 24 h, followed by Western blotting with indicated antibodies. Densitometry for the bands in (**C)** and (**E)** was performed using NIH ImageJ, as shown in (**D)** and (**F)**, respectively. Results are the means ± SE and are pooled from three independent experiments. ^*a*^, *P* < 0.05, difference *verse* control (0 μM CPX). (**G)** MDA-MB-231 cells, infected with lentiviral shRNA to ATR or GFP (control), were incubated with CPX (0-20 μM) for 24 h, followed by Western blotting with indicated antibodies. β-tubulin was used for loading control. (**H)** Densitometry for the bands in (**G)** was performed using NIH ImageJ. Results are the means ± SE and are pooled from three independent experiments. ^*a*^, *P* < 0.05, difference *verse* control (0 μM CPX); ^*b*^, *P* < 0.05, difference between sh-GFP treatment and sh-ATR treatment.

Chk1 can be activated either by ATR in the presence of single-stranded DNA damage [54-56] or by ATM in response to double-stranded DNA damage [57]. Next, we asked whether CPX-induced activation of Chk1 is through ATR and/or ATM. To answer this question, first of all, we checked whether CPX activates ATM and ATR. As shown in Figures 5A and 5B, treatment with CPX (5 µM) for 2-4 h was able to induce remarkable phosphorylation of both ATM and ATR. However, the phosphorylation levels of ATM and ATR declined gradually after 8-h treatment, which was correlated to the decrease in their total protein levels (Figure 5A). Nevertheless, the results indicate that CPX is able to activate both ATR and ATM.

Next, we further determined whether ATM and/or ATR mediates CPX-induced Cdc25A degradation. For ATM, MDA-MB-231 cells were pretreated for 1 h with or without Ku55933 (10 μM), a selective inhibitor of ATM [58], and then exposed to CPX (0-20 μM) for 24 h. We found that CPX (10-20 μM) alone dramatically downregulated Cdc25A expression in the cells; Ku55933 pretreatment did not obviously prevent CPX from reducing Cdc25A expression (Figures 5C and 5D). The result suggests that ATM is not involved in mediating CPX-induced Cdc25A degradation.

For ATR, MDA-MB-231 cells were pretreated with or without caffeine (2 mM), an ATM/ATR inhibitor [59], for 2 h, and then exposed to CPX (0-20 μM) for 24 h, followed by Western blot analysis. As shown in Figures 5E and 5F, treatment with CPX (0-20 μM) alone reduced the expression of Cdc25A in a concentration-dependent manner, which was blocked by caffeine treatment. Since ATM was found not to be involved in mediating CPX-induced Cdc25A degradation (Figures 5C and 5D), the result implies that CPX promotes Cdc25A degradation probably by activation of ATR.

Considering that 2 mM of caffeine may off-target other kinases, to substantiate the role of ATR in mediating Cdc25A degradation, lentiviral shRNA to ATR was used. As shown in Figures 5G and 5H, lentiviral shRNA to ATR effectively silenced the expression of ATR in MDA-MB-231 cells, compared with the control (lentiviral shRNA to GFP). Of interest, knockdown of ATR conferred a high resistance to CPX-induced reduction of Cdc25A protein level. Collectively, these results support that ATR, but not ATM, mediates CPX induced-degradation of Cdc25A protein.

### CPX induces DNA damage, which is related to iron chelation, but not ROS induction

**Figure 6:**
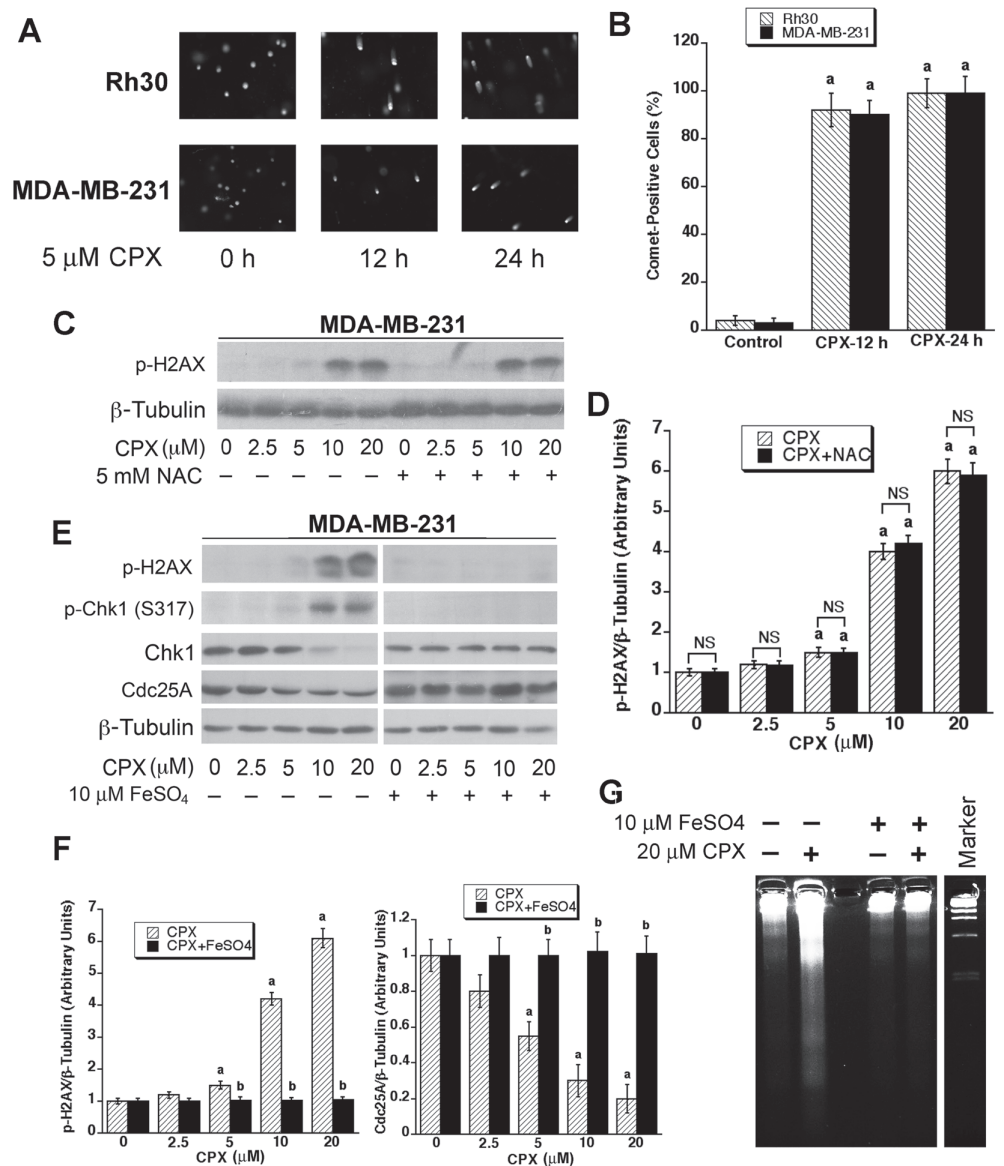
CPX causes DNA damage, which is not related to ROS induction, but due to iron chelation (**A)** and (**B)** Comet assay was performed in MDA-MB-231 and Rh30 cells treated with CPX (5 μM) for 0, 12 h and 24 h, respectively. The results were visualized and photographed under a fluorescence microscope at 494 nm (excitation) and 521 nm (emission). Representative photos are shown in (A), while quantitative results are illustrated in (**B)**. ^*a*^, *P* < 0.05, difference *verse* control (CPX, 0 h). (**C-G)** MDA-MB-231 cells were pretreated with or without NAC (5 mM) (**C)** or ferrous sulfate (10 μM) (**E)** and (**G)** for 2 h, and then incubated with CPX (0-20 μM) for 24 h, followed by Western blotting with indicated antibodies (**C)** and (**E)** or DNA laddering (**G)** Densitometry for the bands in (**C)** and (**E)** was performed using NIH ImageJ, respectively. Results are the means ± SE and are pooled from three independent experiments. ^*a*^, *P* < 0.05, difference *verse* control (0 μM CPX); ^*b*^, *P* < 0.05, difference between CPX group and CPX+FeSO_4_ group. NS, no significance.

As a member of the DNA damage response (DDR) pathway, ATR is frequently activated in response to DNA damage [60]. Hence, we speculated that CPX-induced activation of ATR-Chk1 is associated with DNA damage. To this end, we performed DNA damage analysis. Both MDA-MB-231 and Rh30 cells were treated with CPX (5 μM) for 0-24 h, followed by the comet assay. We found that treatment with CPX (5 μM) for 12-24 h significantly increased the number of cells with the comet tail, a marker of DNA damage, compared to the control (Figures 6A and 6B). The results indicate that 5 μM of CPX is able to cause DNA damage in the cells.

Induction of reactive oxidative species (ROS) is considered a major cause of DNA damage [61]. As we have observed that treatment with CPX can induce ROS in tumor cells [11], next we tested whether CPX-induced DNA damage is related to the induction of ROS. Pretreatment with 5 mM of N-acetyl-L-cysteine (NAC), an antioxidant and ROS scavenger [62], for 2 h, failed to prevent the DNA damage induced by CPX. This is evidenced by the finding that treatment with CPX (0-20 μM) alone induced the phosphorylation on H2AX, a hallmark of DNA damage [63], in a concentration-dependent manner (Figures 6C and 6D). Particularly, at the concentrations of 10-20 μM, CPX was able to induce robust phosphorylation of H2AX. However, pretreatment with NAC did not obviously attenuate CPX-induced phosphorylation of H2AX (Figures 6C and 6D). The results suggest that CPX-induced ROS may not play a dominant role in inducing DNA damage.

Since CPX is an iron chelator [4], and iron chelation can cause DNA damage [64, 65], next, we examined whether CPX causes DNA damage through chelating iron. For this, MDA-MB-231 cells were pretreated with or without ferrous sulfate (10 μM) for 1 h, and then exposed to CPX (0-20 μM) for 24 h, followed by Western blot analysis. Treatment with CPX (0-20 μM) alone elevated the phosphorylation levels of H2AX and Chk1, as well as reduced the protein level of Cdc25A in a concentration-dependent manner. Interestingly, pretreatment with ferrous sulfate remarkably attenuated CPX-induced H2AX/Chk1 phosphorylation and Cdc25A degradation (Figures 6E and 6F). In addition, pretreatment with ferrous sulfate also blocked CPX-induced DNA laddering (DNA damage) (Figure 6G). The data support the notion that the iron chelation activity of CPX contributes to DNA damage, which activates the DDR pathway, thereby leading to Cdc25A degradation.

## DISCUSSION

CPX, an off-patent fungicide, has been used to treat fungal infection of the skin and nails for > 20 years [1, 2]. Recently, it has been shown that CPX also possesses potent anti-proliferative effect on tumor cells [4, 5]. Mechanistically, on one hand, CPX inhibits ribonucleotide reductase (RR), an enzyme that catalyzes the formation of deoxyribonucleotides from ribonucleotides, thus suppressing DNA synthesis [4]. On the other hand, CPX inhibits G_1_-CDKs by downregulating the cellular protein levels of cyclins (A, B1, D1 and E) and cyclin-dependent kinases (CDK2 and CDK4), and upregulating the protein level of the CDK inhibitor (p21^Cip1^) [5]. Recently, we have further observed that CPX inhibits G_1_-CDKs also in part by promoting proteasome-dependent degradation of Cdc25A protein [32]. Here, we present evidence that CPX-induced Cdc25A degradation is attributed to activation of ATR-Chk1 pathway, a consequence of iron chelation-induced DNA damage.

Our previous study has shown that Cdc25A-S82A mutant is resistant to CPX-induced degradation [5], suggesting that the elevated phosphorylation on S82 might result in consequent ubiquitination and degradation. Since S82 of Cdc25A is phosphorylated by CK1α [40], at the beginning, we hypothesized that CPX might upregulate CK1α expression. As the cellular protein level of CK1α determines its activity [46], we directly tested whether CPX increases CK1α protein level. In contrast to our hypothesis, CPX slightly reduced the protein level of CK1α (Figures 1A and 1B). Since Cdc25A degradation is β-TrCP ubiquitin-dependent [41, 66], next, we checked whether CPX downregulates DUB3, a Cdc25A-specific deubiquitinase, which protects Cdc25A from degradation by removing ubiquitin chain from Cdc25A [47]. Again, CPX did not reduce, but unexpectedly increased DUB3 protein expression slightly (Figures 1C and 1D). Currently, we have no clue why and how CPX downregulated CK1α and upregulated DUB3 expression. Possibly, this was a cellular response that tried to rescue Cdc25A from degradation.

The above observations led us to think about whether CPX induces Cdc25A phosphorylation on S82 by promoting the phosphorylation on a prime residue, reportedly S76 [37]. As S76 can be regulated by GSK3β [37, 44] and Chk1 [39, 51], we studied whether GSK3β and Chk1 are involved in the regulation of Cdc25A expression, in response to CPX exposure. Using LiCl, a specific inhibitor of GSK3β [48], we ruled out the involvement of GSK3β (Figure 2). Finally, using pharmacological and genetic inhibition of Chk1 activity, we identified Chk1 as the kinase that was activated by CPX and involved in CPX-induced Cdc25A degradation (Figures 3 and 4). This was substantiated by a further observation that ATR, an upstream kinase of Chk1, was also activated by CPX and involved in CPX-induced Cdc25A degradation (Figure 5). It has been described that activated Chk1 phosphorylates S76 on Cdc25A, priming the further phosphorylation on S79 and S82, by CK1α [37-40]. The phosphorylation of S82 facilitates Cdc25A ubiquitination and subsequent proteasome-mediated degradation [40, 41], inhibiting CDK2 activity [42] and arresting cells at G_1_ phase of the cell cycle [43]. Our previous study has shown that CPX treatment induced the phosphorylation of Cdc25A on S76 and S82 [32]. In this study, we did not observe that CPX upregulated the expression of CK1α or triggered the activation of GSK3, but we did discover that CPX induced the activation of ATR-Chk1 pathway. Collectively, our results support the notion that ATR-Chk1 cascade mediates CPX-induced Cdc25A degradation.

It should be mentioned that although our data suggested that the phosphorylation and activation of Chk1 was mainly attributed to CPX-induced ATR activation, we still cannot rule out other possibilities. For instance, PP2A (protein phosphatase 2A), a serine/threonine protein phosphatase, is capable of dephosphorylating Chk1 [67]. Of importance, PP2A activity depends on cellular iron level [68]. CPX is a well-known iron chelator [4], so we deduce that CPX may inhibit PP2A activity. Further research is needed to address whether PP2A plays a role in CPX-induced Chk1 activation, and Cdc25A degradation.

The cross-identification with the DNA laddering and the comet assay confirmed that CPX was able to cause DNA damage, rationalizing the activation of DDR pathway. During the course of studying how CPX causes the DNA damage, we first thought of ROS, since CPX is able to induce ROS [11], and induction of ROS is a major cause of DNA damage [61]. However, to our surprise, CPX-induced DNA damage was not due to induction of ROS, as NAC, a ROS scavenger, did not have obvious protective effect (Figures 6C and 6D). Instead, CPX caused the DNA damage through its iron chelation activity. This is strongly supported by the finding that addition of ferrous sulfate blocked CPX-induced DNA damage (Figures 6E-6G). A new question from this work is how the iron chelation causes DNA damage. A previous report has shown that RR, an iron-dependent dNTP (deoxynucleotide triphosphates) generator, mediates the genotoxicity of CPX [64]. However, a very high concentration of CPX (100 μM) was used in that study, which was in great contrast to low concentrations (5-20 μM) of CPX we used here. Nevertheless, a recent study has shown that treatment with 24-h exposure to CPX (5-10 μM) can inhibit RR activity in leukemia (MDAY-D2) cells [4]. Hence, the RR is a possible candidate that may mediate the CPX-caused DNA damage, which could be blocked by addition of FeSO_4_. We have already tested whether addition of dNTP, a major product of RR, could attenuate CPX-caused DNA damage. However, addition of dNTP failed to prevent CPX-induced damage. We do not know whether this was due to poor membrane transportation efficiency of dNTP in our cell culture model. In the future, an alternative approach is to examine whether overexpression of M1/M2 subunits (RR) attenuates CPX-induced DNA damage, ATR-Chk1 activation, Cdc25A turnover, cell cycle progression, and apoptosis.

**Figure 7:**
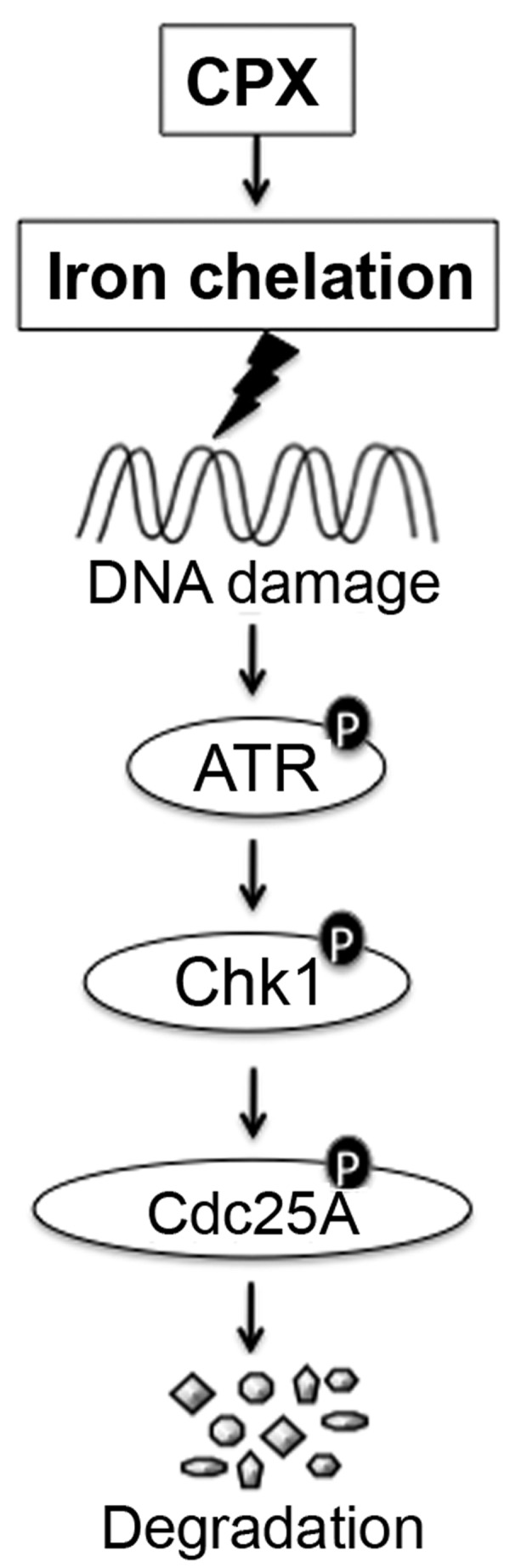
A model of CPX-induced Cdc25A degradation

In conclusion, here we have shown that CPX induced Cdc25A degradation neither by increasing CK1α or decreasing DUB3 expression, nor *via* activating GSK3β in rhabdomyosarcoma (Rh30) and breast carcinoma (MDA-MB-231) cells. Instead, CPX-induced Cdc25A degradation was associated with the activation ATR-Chk1 cascade, resulted from iron chelation-induced DNA damage (Figure 7). The findings shed new light on the molecular mechanism by which CPX inhibits cancer cell proliferation.

## MATERIALS AND METHODS

### Materials

Ciclopirox olamine (CPX) (Sigma, St. Louis, MO, USA) was dissolved in 100% ethanol to prepare stock solutions (2.5, 5, 10, and 20 mM), then aliquoted and stored at −20°C. RPMI 1640, Dulbecco's Modified Eagle Medium (DMEM), DMEM/F-12 and 0.05% Trypsin-EDTA were purchased from Mediatech (Herndon, VA, USA). Fetal bovine serum (FBS) was from Atlanta Biologicals (Lawrenceville, GA, USA). Enhanced chemiluminescence solution was obtained from PerkinElmer Life Science (Boston, MA, USA). The following primary antibodies were used, including those against Cdc25A, Chk1, p-Chk1 (S345), Chk2, p-Chk2 (T68), CK1α, GSK3β (Santa Cruz Biotechnology, Santa Cruz, CA, USA), DUB3 (Abcam, Cambridge, MA, USA), p-GSK3β (S9), p-Chk1 (S317), ATM, p-ATM (S1981), ATR, p-ATR (S428), p-H2AX (S139) (Cell Signaling, Danvers, MA, USA), and β-tubulin (Sigma). Goat anti-mouse IgG-horseradish peroxidase (HRP) and goat anti-rabbit IgG-HRP were purchased from Pierce (Rockland, IL, USA). All other chemicals were obtained from Sigma (St. Louis, MO), unless stated elsewhere.

### Cell culture

Human rhabdomyosarcoma (Rh30) cell line was described [32] and grown in antibiotic-free RPMI 1640 medium supplemented with 10% FBS. Human breast adenocarcinoma (MDA-MB-231) cell line (American Type Culture Collection, Manassas, VA) was grown in antibiotic-free DMEM/F12 supplemented with 10% FBS. 293TD cells were cultured in DMEM containing 10% heat-inactivated FBS, as described [69]. All cell lines were cultured in a humidified incubator (37ºC and 5% CO_2_).

### Lentiviral shRNAs to Chk1 and ATR and infection

Plasmids encoding shRNAs to human Chk1 (sc-29269-SH) and human ATR (sc-44284-SH) were purchased from Santa Cruz Biotechnology (Santa Cruz, CA). The plasmids were amplified in TOP 10 competent cells (Invitrogen, Carlsbad, CA, USA) and extracted with Qiagen Plasmid Midi Kit (Qiagen, Valencia, CA, USA). Finally, lentiviral particles were produced in 293TD cells by co-transfection with shRNA to ATR or Chk1 (10 μg), together with PMD2.0 (3 μg) and psPAX2 (7 μg), as described previously [69]. Cells were seeded in 6-well plates at a density of 1 × 10^5^ per well and cultured under normal condition overnight. The cells were then infected with infection solution (fresh medium : lentiviral particle = 1:1, v/v, plus 5 μM polybrene) and cultured under normal condition. After 12-24 h, the cells were re-infected with the above infection solution and cultured overnight under normal condition. Next, the virus-containing medium was replaced with fresh growth medium containing puromycin (5 μg/ml) for selection of infected cells. After culture for 4 days, the selected cells were used for experiments.

### Western blotting

Cells were briefly rinsed with ice-cold phosphate buffered saline (PBS) and lysed, followed by Western blotting as described previously [32]. NIH ImageJ was used for semi-quantitative analysis of the intensities of the bands.

### DNA laddering

DNA laddering was performed as described [70]. Briefly, MDA-MB-231 cells, grown in 6-well plates (5 × 10^5^ cells/well), were treated with pretreated with/without 10 μM of ferrous sulfate (FeSO_4_) for 1 h, and then treated with/without 20 μM of CPX for 24 h. Both floating and attached cells were harvested, washed with ice-cold 1× PBS, and treated with the lysis buffer (50 mM Tris, pH 8.0, containing 0.5% Triton X-100 and 200 mM EDTA) (200 μl/well) on ice for 20 min. After centrifuging at 12,000 rpm for 30 min at 4°C, the supernatants were transferred into new Eppendorf tubes, and mixed with the same volume of phenol:chloroform:isoamyl alcohol (25:24:1, v/v) (Invitrogen) for 5 min. Following centrifugation (12,000 rpm, 10 min, 4C), the supernatants were transferred to new tubes, mixed with half volumes of ammonium acetate (10 M) and 2.5× volumes of cold ethanol (-20°C), and then incubated at -20°C overnight. After centrifuging at 16,000 rpm for 10 min, the supernatants were carefully discarded, and the precipitates were resuspended in 20 μl of TE buffer-RNase A solution to dissolve DNA (37°C for 30 min). The concentration of the extracted DNA was quantified by NanoDrop 1000 (Nano Drop, Wilmington, DE, USA). The DNA samples were separated onto a 2% agarose gel (containing 0.5 µg/ml ethidium bromide) in TBE running buffer. The images were visualized and captured with a UV transilluminator equipped with a digital camera.

### Comet assay

Comet assay was conducted as described [71]. Briefly, low melting point agarose (0.5 g in 50 ml PBS) was put in a 100-ml glass bottle (cap loosened), and incubated in a beaker containing boiling water until the agarose was melted. The bottle was then placed in a 37°C water bath for at least 20 min to cool down the agarose. Subsequently, freshly trypsinized cells (1 × 10^5^/ml) were mixed with the melted agarose (at 37°C) at a ratio of 1:10 (v/v), and 50 μl of the mixture was immediately pipetted onto 96-well CometSlide™ (Trevigen, Gaithersburg, MD, USA). After placing the slides flat at 4°C in the dark for 10-30 min, the slides were immersed in pre-chilled Lysis Solution (Trevigen) and left at 4°C for 1 h. Next, the slides were removed from the Lysis Solution, and immersed in pre-chilled 1 Tris/Borate/EDTA (TBE) buffer at 4°C for 30 min. Then, the slides were aligned equally distant from electrodes. Subsequently, 1× TBE buffer was added not to exceed 0.5 cm above the slides, followed by electrophoresis (at 1 volt per cm). After electrophoresis, the slides were immersed in distilled water for 5 min at room temperature, and then in 70% ethanol for 30 min at room temperature. After the slides were air-dried at room temperature for 10-15 min, 100 μl of diluted SYBR Green I was placed onto each sample and incubated for 30 min. Next, the slides were completely dried at room temperature in the dark. Finally, the slides were visualized and photographed under a Nikon epifluorescence microscope at 494 nm (excitation) and 521 nm (emission).

### Statistical analysis

Results were expressed as mean values ± standard errors (mean ± SE). The data were analyzed by one-way analysis of variance (ANOVA) followed by post-hoc Dunnett's *t*-test for multiple comparisons. A level of *P* < 0.05 was considered to be statistically significant.

## Abbreviations

ATM: ataxia telangiectasia mutated
ATR: ATM-and RAD3-related
Cdc25A: cell division cycle 25 A
CDK: cyclin-dependent kinase
CK1α: casein kinase 1α
CPX: ciclopirox olamine
DDR: DNA damage response
DMEM: Dulbecco's Modified Eagle Medium
dNTP: deoxynucleotide triphosphates
FBS: fetal bovine serum
GFP: green fluorescence protein
GSK3β: glycogen synthase kinase 3β
NAC: N-acetyl-L-cysteine
PBS: phosphate buffered saline
ROS: reactive oxygen species
PP2A: protein phosphatase 2A
RR: ribonucleotide reductase
